# Natural Products from Chilean and Antarctic Marine Fungi and Their Biomedical Relevance

**DOI:** 10.3390/md21020098

**Published:** 2023-01-29

**Authors:** Dioni Arrieche, Jaime R. Cabrera-Pardo, Aurelio San-Martin, Héctor Carrasco, Lautaro Taborga

**Affiliations:** 1 Laboratorio de Productos Naturales, Departamento de Química, Universidad Técnica Federico Santa María, Avenida España 1680, Valparaíso 2340000, Chile; 2 Laboratorio de Química Aplicada y Sustentable (LabQAS), Departamento de Química, Universidad del Bio-Bio, Avenida Collao 1202, Concepción 4030000, Chile; 3 Departamento de Ciencias y Recursos Naturales, Facultad de Ciencias Naturales, Universidad de Magallanes, Avenida Bulnes 01855, Punta Arenas 6200112, Chile; 4 Grupo QBAB, Instituto de Ciencias Químicas y Aplicadas, Facultad de Ingeniería, Universidad Autónoma de Chile, Llano Subercaseaux 2801, Santiago 8900000, Chile

**Keywords:** marine natural products, marine fungi, Chilean marine fungi, biological activities

## Abstract

Fungi are a prolific source of bioactive molecules. During the past few decades, many bioactive natural products have been isolated from marine fungi. Chile is a country with 6435 Km of coastline along the Pacific Ocean and houses a unique fungal biodiversity. This review summarizes the field of fungal natural products isolated from Antarctic and Chilean marine environments and their biological activities.

## 1. Introduction

Natural products (NPs) represent a rich and vast biologically relevant chemical space that remains extremely difficult to access with the current arsenal of tools in chemical synthesis [1]. NPs are characterized by enormous scaffold diversity and structural complexity. Nature, via evolution, has optimized secondary metabolites to serve pivotal biological functions, including endogenous defense mechanisms as well as interaction with other organisms [2]. Natural products-based medicines can be traced back thousands of years and still contribute to many approved drugs. Indeed, natural products and their derivatives represented 27% of all therapeutics approved by the FDA between 1981 and 2019 [3,4]. In recent years, this proportion has increased, illustrating the continued importance of NPs. Research programs focused on unveiling new NPs from understudied microorganisms, such as fungi isolated from Chilean marine environments, are crucial to the future drug development pipeline.

Fungi represent one of the largest groups of organisms. They are widely distributed across both mild and extreme ecosystems on our planet [5]. They have developed a unique metabolic plasticity, allowing them to rapidly adapt and survive through the biosynthesis of an array of fascinating natural products [6]. A recent analysis of fungal genomes has revealed many secondary metabolite pathways that can be tuned or modified, producing novel and valuable chemical scaffolds [7]. Fungal-derived natural products are pharmaceutically abundant, with several important biological applications ranging from highly potent toxins to approved drugs [8]. Since the discovery of penicillin, an antibiotic of fungal origin, many efforts around the globe have been devoted to searching for fungal-derived bioactive products. Fungi are a vast yet untapped source to search for pharmaceutically relevant molecules displaying a range of bioactivity, including anti-cancer, antioxidant, hepatoprotective, antibacterial, antidiabetic, and anti-inflammatory capabilities.

Oceans are the source of a wide variety of natural products with unique structures mainly produced by marine macro-organisms, such as invertebrates (e.g., sponges, soft corals, tunicates) and algae. Additionally, many marine natural products have proved to be pharmacologically relevant [9,10,11].

Secondary metabolites obtained from marine fungi have been particularly interesting, mainly because of their unique chemical structures and biomedical applications [8,12]. In 1949, cephalosporin C was discovered from a culture of *Cephalosporium* fungus species obtained from the Sardinian coast [13]. Since then, extensive efforts over decades of work have revealed the vast chemical and biological potential of marine fungal natural products. Strains of marine fungi have been obtained from practically every possible marine habitat, including inorganic, marine microbial communities, marine plants, and marine vertebrates [10]. While the number of cultivable marine fungi is extremely low (1% or less) compared to their global biodiversity [8,9,10], the number of natural products that have been isolated and characterized from marine fungi exceeds 1000 molecules [14]. These include alkaloids, lipids, peptides, polyketides, prenylated polyketides, and terpenoids [14,15,16,17].

Chile has 6435 km of coastline and exercises exclusive rights over its maritime space called the Chilean Sea. This comprises four zones: the Territorial Sea (120,827 km^2^), the contiguous zone (131,669 km^2^), the exclusive economic zone (3,681,989 km^2^), and that corresponding to the Continental shelf (161,338 km^2^) [18].

The Chilean maritime territory, in the Pacific Ocean, consists of highly structured geographic sections displaying unique features that arise from the interactions of water masses with the seabed, emerged relief, air masses and centers of atmospheric action [19]. These phenomena lead to an environment suitable for a rich biodiversity ranging from microscopic organisms that swarm the waters in incredible numbers to large fish and other organisms [20]. Along the lengthy coastline, Chilean waters also differ in terms of important characteristics, e.g., mineral and saline composition [19].

The cold waters associated with both the Humboldt and Antarctic currents are characterized by a high gas and nitrogen content, unlike in temperate and warm waters. Consequently, phytoplankton is abundant in the Chilean sea and supports the growth of various marine organisms, specifically fungi. Therefore, Chile´s coastline provides a distinct environment for fungal biodiversity to flourish [21]. 

Despite its importance, there are not many reports about secondary metabolites from marine fungi or marine-derived fungi in Chile. Reports included in this review cover the period from 1996 until present. In this work, we have made a comprehensive review of compounds that have been isolated and chemically characterized during this time. Their biological activities are also reported.

## 2. Secondary Metabolites Isolated from Chilean Marine Fungi in Continental Coasts

**Figure 1:**
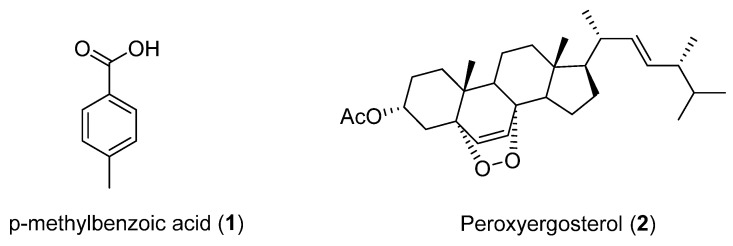
Secondary metabolites isolated from *Cladosporium cladosporioides*.

Studies carried out on cultures of *Cladosporium cladosporioides*, a fungus isolated from the marine sponge *Cliona* sp. collected in Region IV of Chile in 2004, led to the identification of *p*-methylbenzoic acid (**1**) and peroxyergosterol (**2**) (Figure 1). This was the first time that **1** had been isolated as a natural product. It was reported that peroxyergosterol from *Inonotus obliquus* inhibited the growth of cancer cells and showed cytotoxic effects on the same cell lines. Additionally, peroxyergosterol displayed potent inhibition of lipid peroxidation and higher antioxidant activity than well-known antioxidants, such as α-tocopherol and thiourea. A recent study also revealed inhibitory effects of peroxyergosterol on inflammation and tumor promotion in mouse skin [22]. In addition, compounds **1** and **2** did not show antimicrobial activity against Gram-positive (*Staphylococcus aureus, S. epidermidis*) or Gram-negative bacteria (*Escherichia coli, Proteus mirabilis*, *Enterococcus faecalis*) in the agar plate diffusion assay. Both compounds were inactive against *Artemia salina* [23].

**Figure 2:**
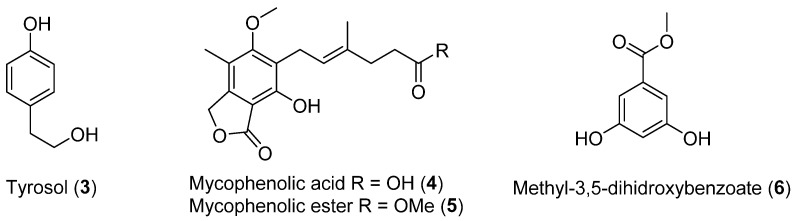
Secondary metabolites isolated from *Penicillium brevicompactun*.

Four previously reported metabolites (**3**–**6**, Figure 2) were isolated from *Penicillium brevicompactun*, collected in Quintay, Chile (region V). The mycelium and broth were extracted with ethyl acetate, and the solvent was evaporated to provide a crude extract that showed in vitro antibacterial activity against both Gram-positive (*Staphylococcus aureus*, *S. epidermidis*) and Gram-negative bacteria (*Escherichia coli*, *Proteus mirabilis*, *Enterococcus faecalis* and *Pseudomonas aeruginosa*) [24].

**Figure 3:**
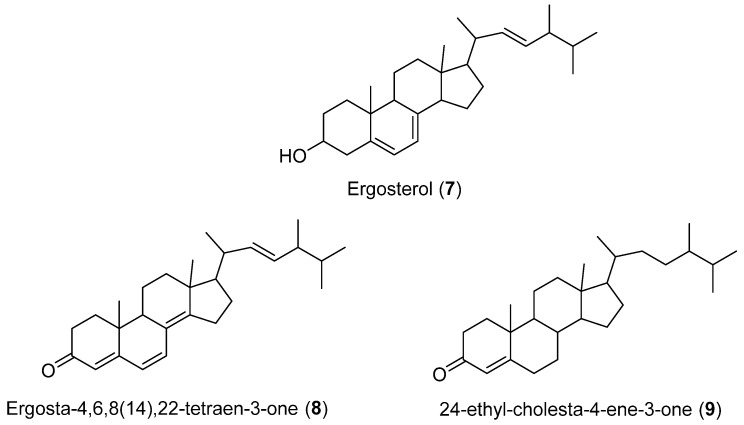
Secondary metabolites isolated from *Geotrichum* sp.

Four steroids (**2**, **7**–**9**) (Figure 1 and Figure 3) were isolated from cultures of *Geotrichum* sp., a fungus obtained from marine sediment collected in Concepción Bay, Chile (Region VIII). Compound **7** is commonly found in fungal extracts since it plays a structural role in the cytoplasmic membrane. Similarly, **2** is a ubiquitous NP present in a variety of lichens, fungi, sponges, and marine organisms. Compound **8** has been isolated from *Lampteromyces japonicus* and a luminous bacterium. Additionally, **8** has been found in non-luminous basidiomycete fungi, including *Fomes officinalis* and *Scleroderma polyrhizum*. It has also been isolated from a marine sponge *Dictyonella incisa* [25]. This is the first time this compound has been identified in a facultative marine fungus [26].

**Figure 4:**
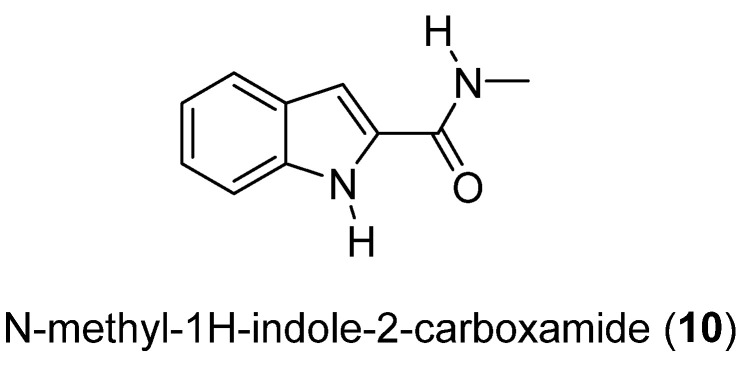
Secondary metabolite isolated from *Cladosporium cladosorioides*.

Compound **10** is the first indole derivative isolated from a marine fungus (*Cladosporium cladosorioides)*. The crystal structure of N-methyl-1H-indole-2-carboxamide (**10**) (Figure 4) was determined by single-crystal X-ray diffraction [27].

**Figure 5:**
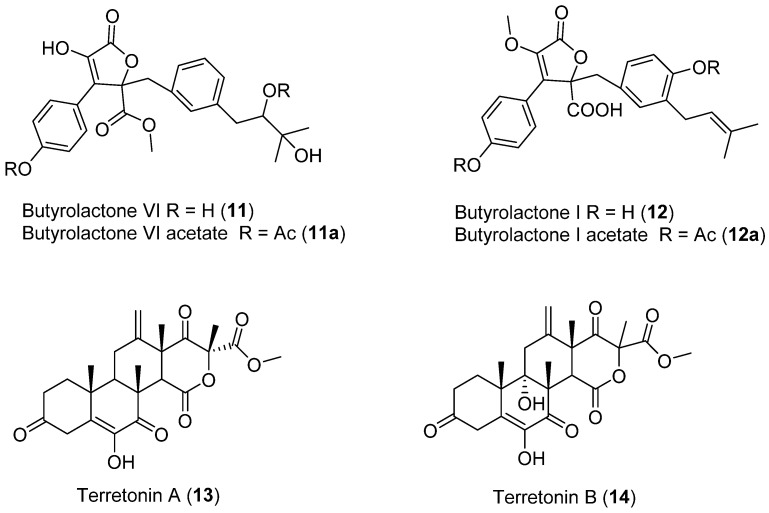
Secondary metabolites isolated from *Aspergillus* sp.

Two dibenzylbutyrolactones (**11**,**12**) (Figure 5) and two sesterterpenoids (**13**,**14**) (Figure 5) were obtained from *Aspergillus* sp. (2P-22) isolated from the marine sponge, *Cliona chilensis* collected in Los Molles, Chile (Region IV) [28]. Spectroscopic data highlighted compound **11** as a novel compound named butylrolactone-VI. All four compounds were then tested for antibacterial activity against both Gram-positive (*Clavibacter michiganensis* 807) and Gram-negative bacteria (*Pseudomonas syringae pv syringae, Xanthomonas arboricola pv juglandis* 833, *Erwinia carotovora, Agrobacterium tumefaciens* A348), vasorelaxant effects, and antitumor bioactivities employing a broth culture of *A. tumefaciens* [28].

## 3. Secondary Metabolites Isolated from Antarctic Marine Fungi

The Antarctic continent represents one of the most extreme environments on earth for life to exist [29]. This ecosystem is characterized by high-stress conditions, including low temperatures, scarce availability of nutrients, high acidity, and high levels of ultra-violet radiation [30]. In order to survive under these highly demanding conditions, fungi living in the Antarctic have had to adapt their biochemical machinery and have done so through modifications in gene expression as well as the biosynthesis of secondary metabolites. Thus, Antarctic fungi represent a unique, biologically relevant chemical space with tremendous potential to contribute to the development of effective therapeutics [31]. Indeed, a number of efforts have reported unique NPs isolated from fungi living in Antarctic environments [31] and this emerging field promises a vast capacity for expansion.

**Figure 6:**
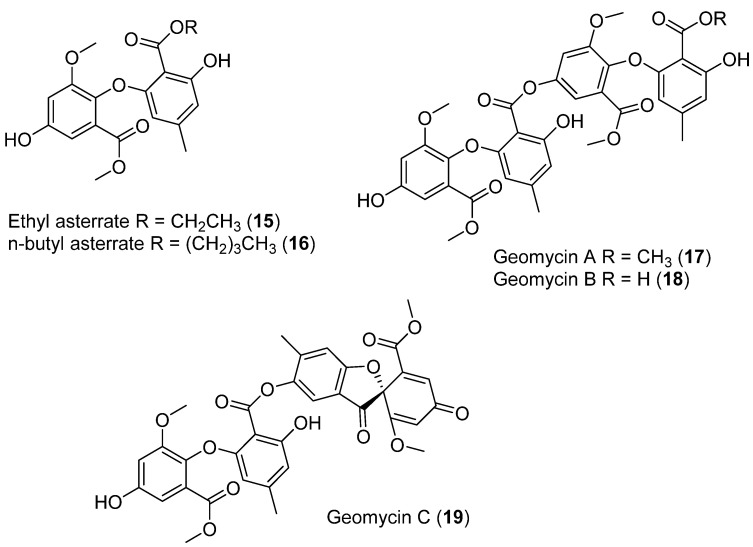
Secondary metabolites isolated from *Geomyces* sp.

In cold marine ecosystems, the presence of fungi has been associated with macroalgae and invertebrates, although some species have also been recorded in seawater and sediments [32,33]. Five new asterric acid derivatives were identified and isolated from the fermentation of the Antarctic ascomycete *Geomyces* sp.: ethyl asterrate (**15**) (Figure 6), n-butyl asterrate (**16**) (Figure 6), and geomycins A–C (**17**–**19**) (Figure 6). These compounds were evaluated for antifungal and antibacterial properties. Geomycin B (**18**) showed significant activity against *Aspergillus fumigatus* ATCC 10894, with IC_50_/MIC values of 0.86/29.5 µM, indicating much higher antifungal activity than the positive control fluconazole, which showed IC_50_/MIC values of 7.35/163.4 µM [31,34].

**Figure 7:**
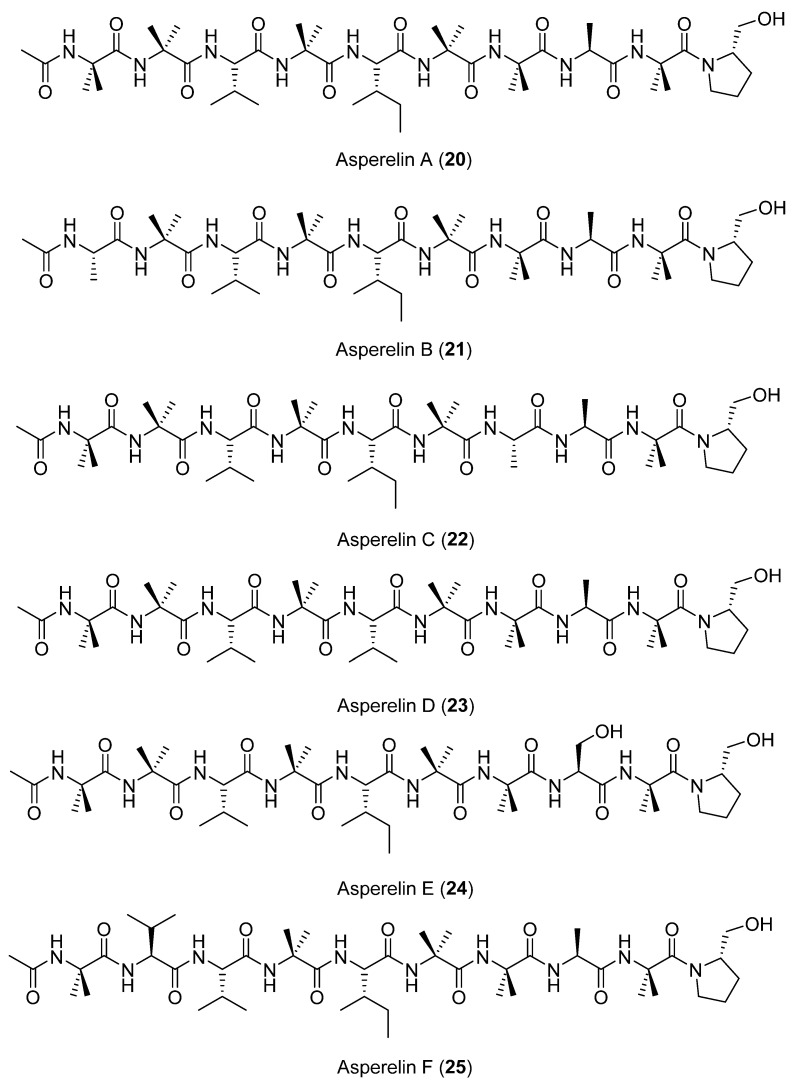
Secondary metabolites isolated from *Tridocherma asperellum*.

Six new peptaibols (linear or cyclic peptides), named asperelines A–F (**20**–**25**) (Figure 7), were characterized from the fermentation of the marine-derived fungus *Tridocherma asperellum* collected from the sediment of the Antarctic Penguin Island. Chemical structures were determined using 1D and 2D NMR techniques as well as ESIMS/MS [35].

**Scheme 1:**
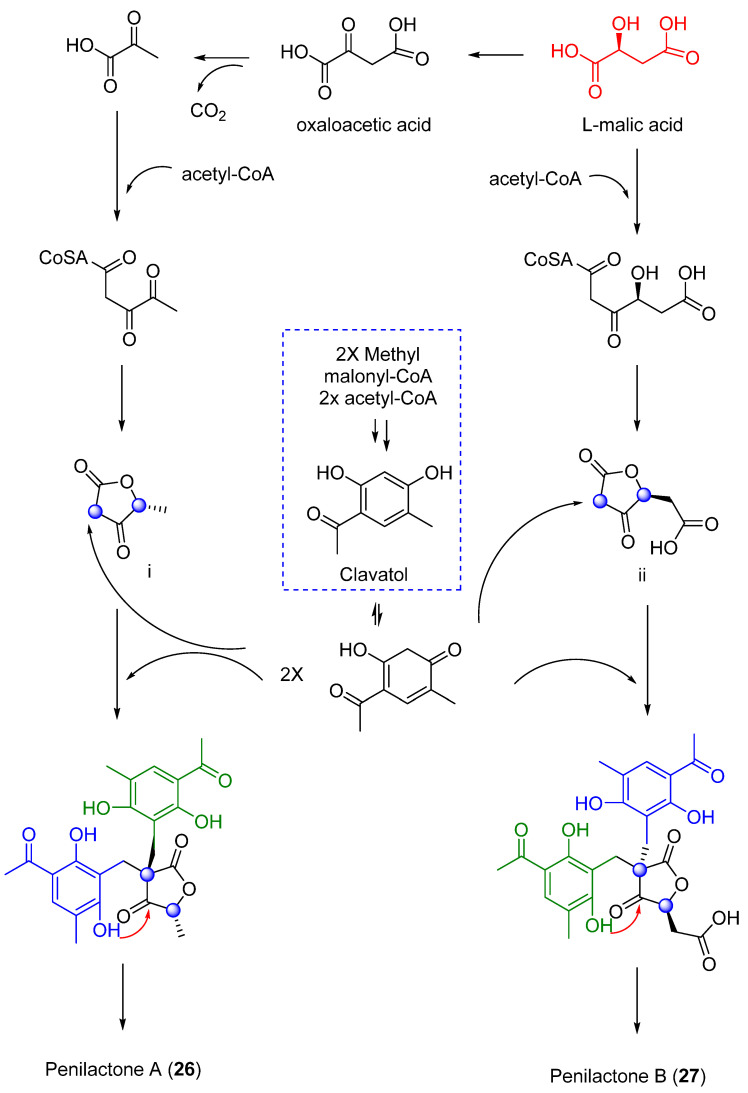
Proposed biosynthetic pathway to **26** and **27**.

**Scheme 2:**
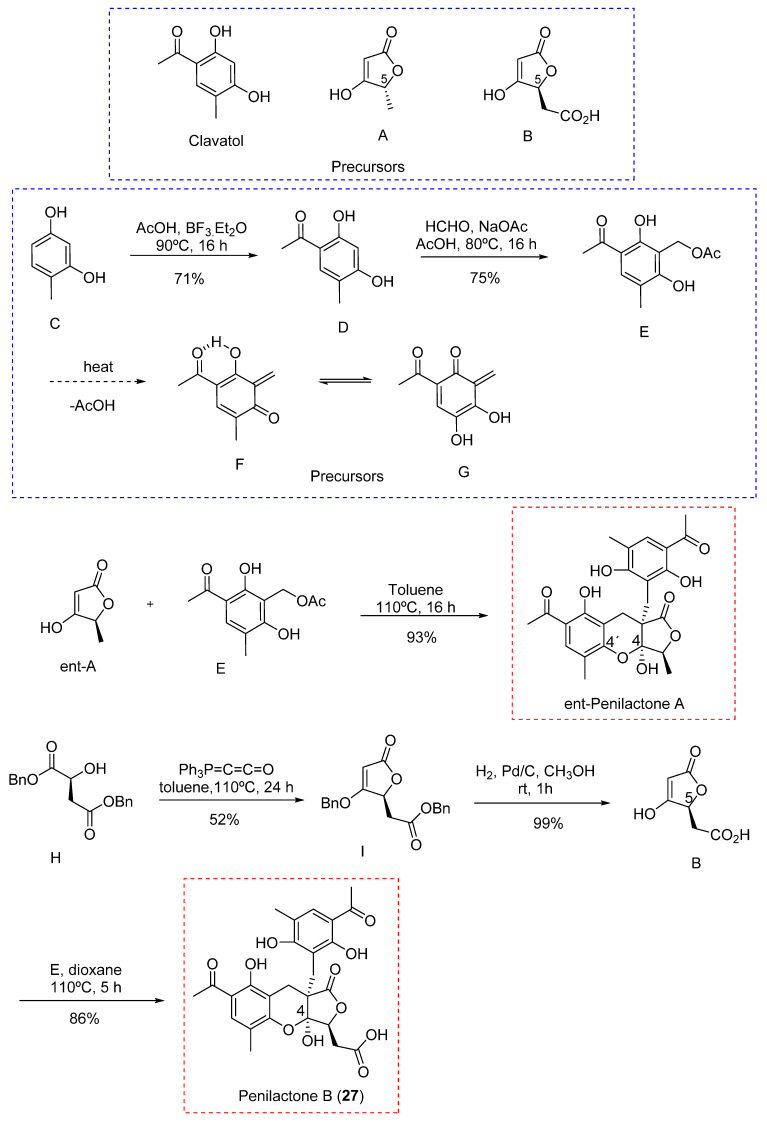
Biomimetic synthesis of *ent*-Penilactone A and Penilactone B.

**Figure 8:**
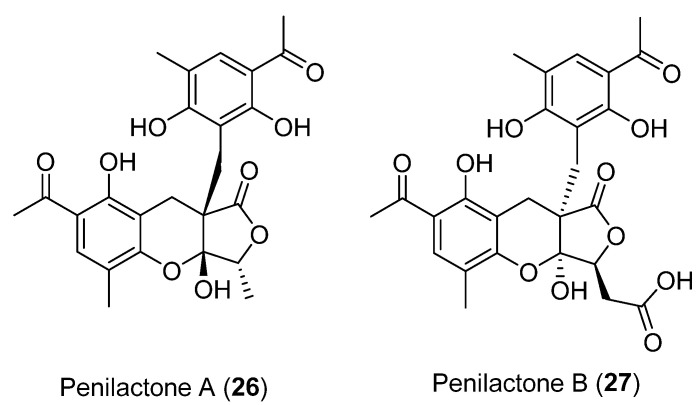
Secondary metabolites isolated from *Penicillium crustosum* PRB-2.

Next, two highly oxygenated polyketides, penylactones A and B (**26** and **27**) (Figure 8) were isolated and identified from *Penicillium crustosum* PRB-2. These compounds had a similar chemical structure but opposite absolute stereochemistry. Compounds **26** and **27** were tested for their ability to inhibit nuclear factor-κB (NF-κB) via transient transfection and reporter gene expression assays. Of the two compounds, only **27** showed inhibitory activity with a relatively weak effect of 40% inhibitory rate at a concentration of 10 µM [36]. The authors also proposed a biosynthetic pathway for both compounds, shown in Scheme 1 [36]. These penylactones are characterized by a new carbon skeleton formed from two units of 3,5-dimethyl-2,4-diol-acetophenone and γ-butyrolactone. Six compounds were subsequently synthesized through a novel biomimetic synthesis pathway, as shown in Scheme 2 [37].

**Figure 9:**
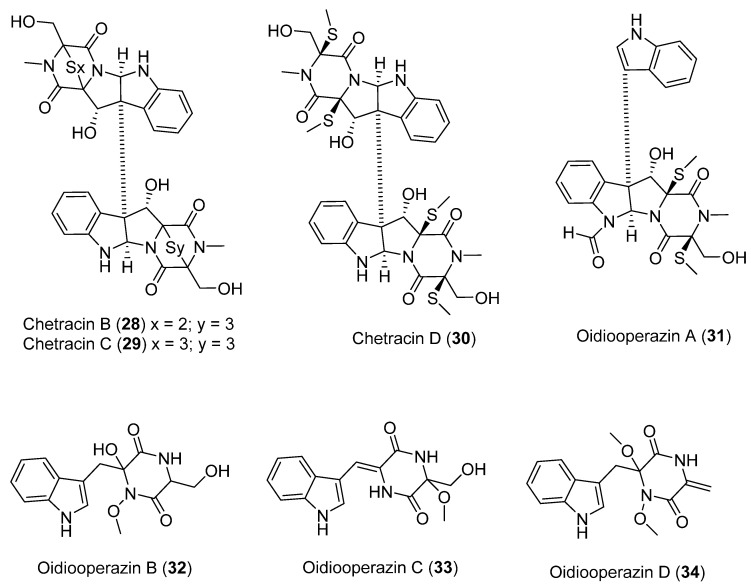
Secondary metabolites isolated from the Antarctic fungus *Oidiodendron truncatum*.

A study of the Antarctic fungus *Oidiodendron truncatum* GW3-13 isolated two new epipolythiodioxopiperazines, chetrazins B (**28**) and C (**29**), together with five new diketopiperazines, chetracin D (**30**), and oidiooperazines A–D (**31**–**34**) (Figure 9). In vitro studies using 3-(4,5-dimethylthiazol-2-yl) 2,5-diphenyltetrazolium bromide (MTT) showed that compound **28** exhibits potent biological activity in the nanomolar range against a panel of five human cancer lines (HCT-8, BEL-7402, BGC-823, A-549, and A-2780) [38].

In the same study, compounds **29** and **30** exhibited significant cytotoxicity at micromolar concentration. Finally, it was observed that compounds **31**–**34** did not show cytotoxicity at a concentration of 10 µM. This led to the conclusion that the sulfide bridge was a determining factor in the biological activity presented by these compounds. In contrast, the number of sulfur atoms in the bridge did not seem to influence the bioactivity [38].

Organic extracts of several fungi were isolated from samples of Porifera collected on King George Island. Although pure compounds could not be isolated, the presence of biological activities and potential as antimicrobial agents could be investigated. Antimicrobial activity was tested using strains of *Pseudomonas aeruginosa*, *Staphylococcus aureus* ATCC25922, *Clavibacter michiganensis* 807, and *Xanthomonas campestris* 833. Antitumoral activity was assessed using *Agrobacterium tumefaciens* At348 as a model, and antioxidant activity was determined by comparing the absorbance of ascorbic acid obtained from each extract. Approximately 50% of the 101 extracts showed antibacterial activity against at least one of the bacteria tested, being more active against Gram-positive bacteria such as *Staphylococcus aureus*. Moreover, 43 extracts showed 50% inhibition of crown gall tumor growth on potato. Antioxidant studies revealed that 97 fungal extracts displayed decent activities varying from very low to mild, and only three isolates showed high antioxidant activities [39].

**Figure 10:**
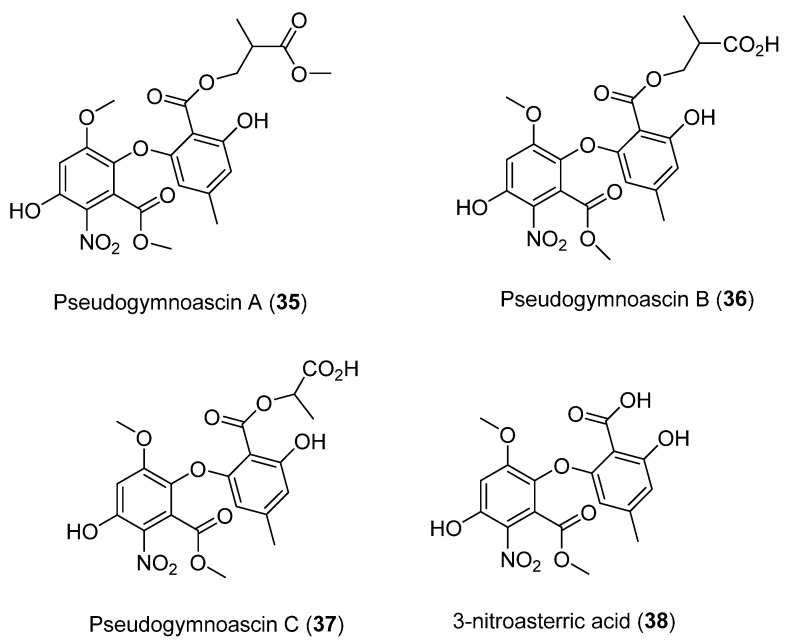
Secondary metabolites isolated from *Pseudogymnoascus* sp.

Four new compounds, namely Pseudogymnoascins A–C (**35**–**37**) and 3-nitroasterric acid (**38**) (Figure 10), were characterized from a culture of *Pseudogymnoascus* sp., obtained from an Antarctic marine sponge of the genus *Hymeniacidon* [40]. Remarkably, these compounds were the first nitro derivatives of asterric acid identified. The antimicrobial activity of compounds **35**–**38** was evaluated against *Pseudomonas aeruginosa* PAO1, *Actinobacter baumannii* CL5973, *Escherichia coli* MB2884, *Staphylococcus aureus* EP1167, and *S. aureus* MB5394. Their antifungal activity was also tested against *Candida albicans* MY1055, *C. albicans* ATCC64124, and *Aspergillus fumigatus* ATCC46645. Cytotoxicity against the tested microorganisms was not observed, suggesting that the presence of the nitro group in the structure may negatively influence the biological activity of these compounds [40].

One hundred fungal strains were isolated from 55 samples of maritime Antarctic and classified into 35 fungal taxa within 20 genera. Extracts from these strains were tested against human tumoral cells, parasitic protozoa (*Leishmania amazonensis*, *Trypanosoma cruzi*), fungi, and bacteria. The extracts from *Purpureocillum lilacinum* displayed high trypanocidal, antibacterial, and antifungal activities with moderate toxicity over normal cells [41].

**Figure 11:**
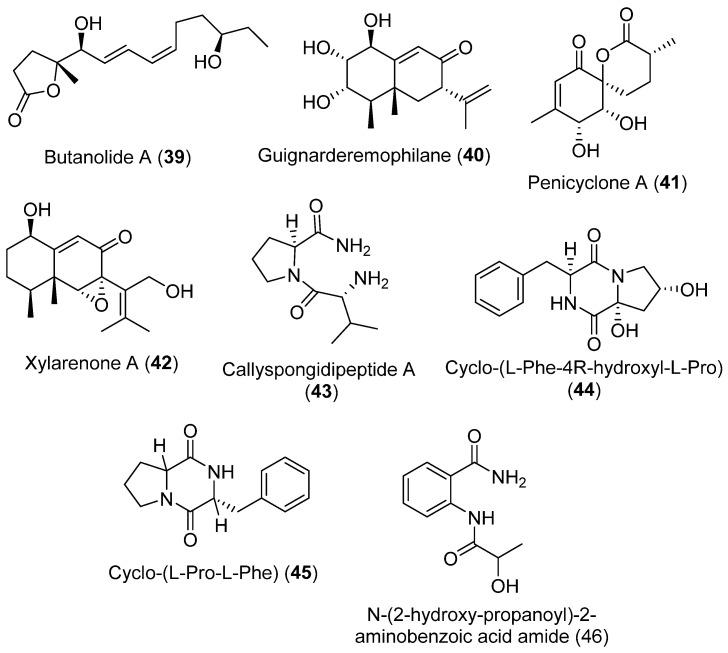
Secondary metabolites isolated from *Penicillium* sp. S-1-18.

In recent years the chemical compounds of *Penicillium* sp. S-1-18 isolated from Antarctic seabed sediments has been extensively investigated. Butanolide A (**39**), a new furanone derivative, and guignarderemophilane F (**40**), a new sesquiterpene, together with six known compounds: penicyclone A (**41**), xylarenone A (**42**), callyspongidipeptide A (**43**), cyclo-(L-Phe-4R-hydroxyl-L-Pro) (**44**), cyclo-(L-Pro-L-Phe) (**45**), and N-(2-hydroxy-propanoyl)-2-aminobenzoic acid amide (**46**), were isolated (Figure 11). The structures of these metabolites were determined using 1D- and 2D-NMR spectroscopic methods. Inhibitory effects against PTP1B activity were tested for all compounds. Only compound **39** showed activity against PTP1B, which was moderate compared with the positive control oleanolic acid [42].

An interesting study of the distribution of marine fungi found that *Pseudogymnoascus* sp. and species of the genus *Penicillium* were present in all marine samples. Samples collected at 20 m or more in depth, at temperatures near 0 °C, had higher diversity those from the intertidal zone (superficial samples) [43].

**Figure 12:**
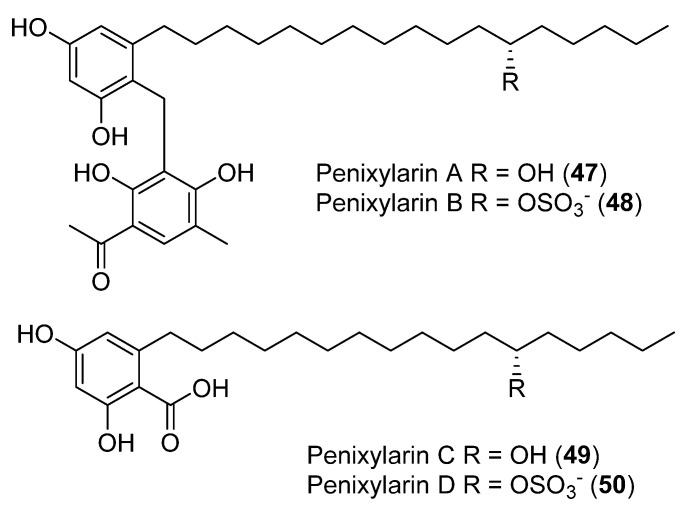
Penixylarins A–D, isolated from *Penicillium crestosum* PRB-2 and the fungus *Xylaria* sp. HDN12-249.

The antibacterial activity was assessed for four new compounds, Penixylarins A–D (**47**–**50**), obtained from a culture of the Antarctic fungus *Penicillium crustosum* PRB-2 and the mangrove-derive fungus *Xylaria* sp. HDN12-249 (Figure 12). Compounds **48** and **49** showed antibacterial activity against *B. subtilis*, *M. phlei*, and *V. parahemolyticus*. Compound **49** additionally displayed potential antituberculosis effects against *Mycobacterium phlei* [44]. 

In a 2018 review, Tripathi et al. described more than two hundred natural products isolated from prokaryotes and eukaryotes living in polar regions, including fungi. Their pharmacology, relevant bioactivity, and chemical structures were reported in the review [45]. One year later, anticancer compounds were isolated from seaweed-derived endophytic fungi [46].

**Figure 13:**
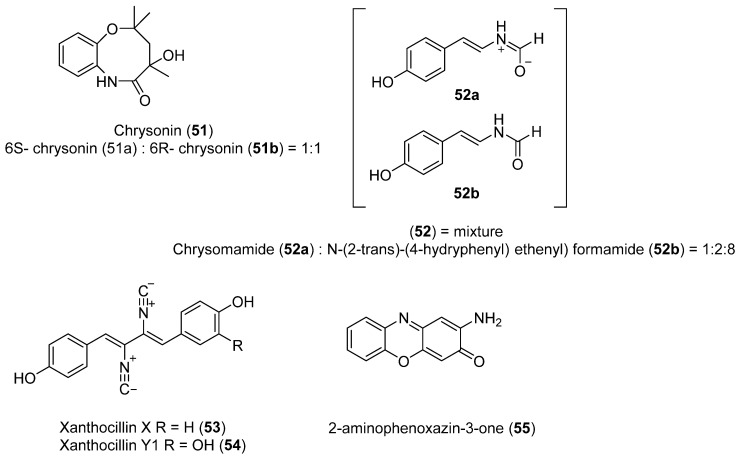
Secondary metabolites isolated from *Penicillium chrysogenum*.

Using a different sampling strategy, pieces of excrement from Adelie penguins allowed the isolation of *Penicillium chrysogenum*. Although the sample was not collected from a marine environment per se, the feeding habits of the penguins support the idea that the microorganisms isolated are marine. The exact location of the sample collection site was not stated but is presumably near the Chinese Great Wall Antarctic base. A new compound Chrysonin (**51**) was obtained as a pair of enantiomers 6S- and 6R-chrysonin (**51a** and **51b**) (Figure 13). These compounds display an eight-membered heterocycle fused with a benzene ring. Interestingly, there is no precedent of one natural compound with this structure. Compound 52 was also isolated as a mixture of a new zwitterionic compound chrysomamide (**52a**) and N-[2-trans-(4-hydroxyphenyl) ethenyl] formamide (**52b**) (Figure 13). Compound **53**, shown in Figure 13, contains the unusual isocyanide functional group. This functional group has been found in several marine organisms, such as cyanobacteria, Penicillium fungi, marine sponges, and nudibranchs. Furthermore, there is no precedent of one natural compound with an eight-membered heterocycle fused with a benzene ring. Antibacterial activity of each compound against eight microorganisms was determined. Compound **53** (Figure 13) was active against *Pseudomonas aeruginosa, Klebsiella pneumoniae*, and *Acinetobacter baumannii*. The same metabolite and compound **54** (Figure 13) both showed significant cytotoxicity against four cancer cell lines: *A. baumannii* ATCC 19606, *E. coli* ATCC 25922, *M. luteus* SCSIO MLO1, and MRSA, shhs-A1. Compound **55** (Figure 13) displayed the best alpha glucosidase inhibition [47]. 

**Figure 14:**
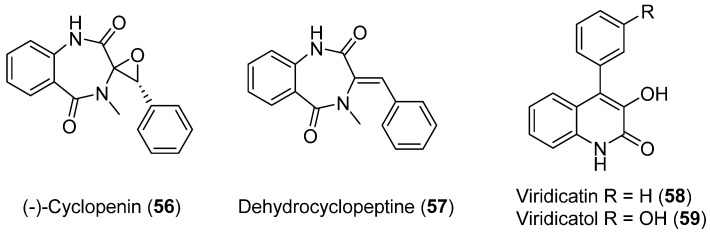
Alkaloids isolated from *Penicillium echinulatum*.

*Penicillium echinulatum* was isolated from the surface of the alga *Adenocystis utricularis* collected on a beach close to Comandante Ferraz Brazilian station on King George Island. In this study, photosafety was evaluated using photoreactivity (OECD TG 495) and phototoxicity assays performed by 3T3 neutral red uptake (3T3 NRU PT, OECD TG 432) and the RHS model. The purification of four alkaloids was achieved in a bio-guided process. Four known metabolites were identified: (−)-cyclopenin (**56**), dehydrocyclopeptine (**57**), viridicatin (**58**), and viridicatol (**59**) (Figure 14), and their photoprotective and antioxidant activities were shown [48].

**Figure 15:**
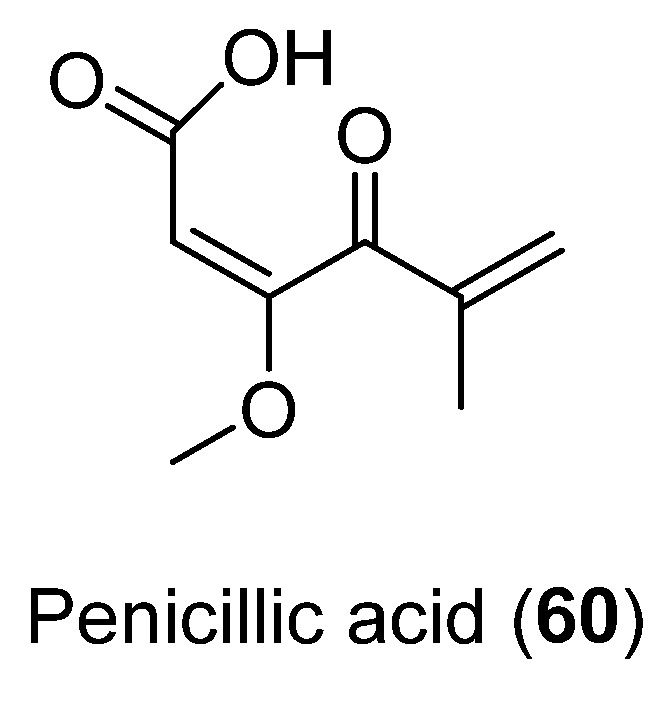
Penicillic acid isolated from *Penicillium* sp. *CRM-1540*.

The antibacterial activity of Penicillic acid (**60**) (Figure 15), isolated from *Penicillium* sp. CRM-1540 found in Antarctic marine sediment at King George Island, was evaluated. This compound was obtained as the major bioactive fraction through a bioguided study. Results showed 90% bacterial inhibition in vitro at 25 µg mL^−1^ against *Xanthomonas citri* [49].

**Figure 16:**
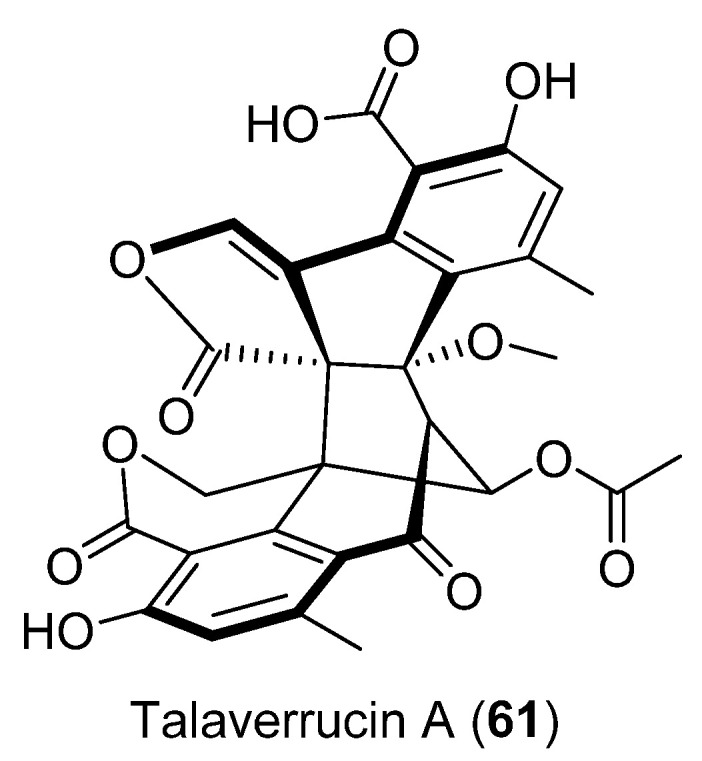
Talaverrucin A isolated from *Penicillium* sp. CRM-1540.

Talaverrucin A (**61**) (Figure 16), a heterodimeric oxaphenalenone with a rare fused ring system, was isolated from *Talaromyces* sp. HDN151403 (Prydz Bay, Antarctica). The oncogenic Wnt/β-catecin inhibitory effect was tested and showed inhibitory activity in zebrafish embryos in vivo and cultured mammalian cells in vitro [50].

**Figure 17:**
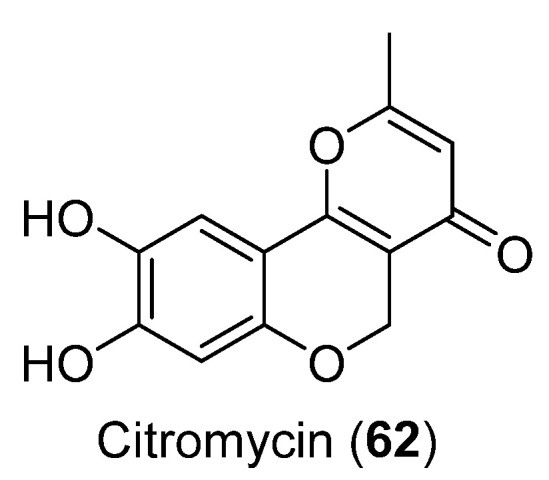
Citromycin isolated from *Sporothrix* sp.

The cytotoxic activity of Citromycin (**62**) (Figure 17) was tested against ovarian cancer SKOV3 and A2780 cells. No cytotoxic activity was observed. The compound **62** was obtained from *Sporothrix* sp. and showed inhibition of extracellular signal-regulated kinase (ERK)-1/1 [51].

**Figure 18:**
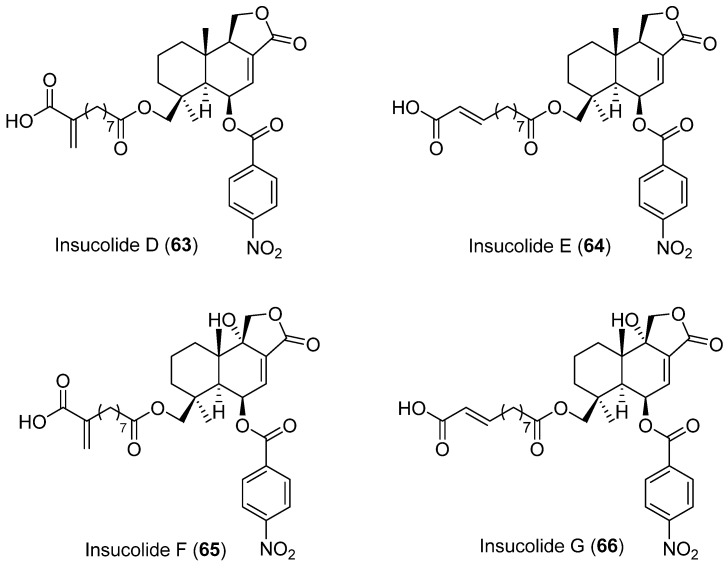
Insucolides (D–G) isolated from *Aspergillus insulicola* HDN151418.

Four new cytotoxic nitrobenzoyl sesquiterpenoids, insulicolides D–G (**63**–**66**) (Figure 18), were isolated from *Aspergillus insulicola* HDN151418, which was obtained from an unidentified Antarctica sponge (Prydz Bay). Compounds **65** and **66** showed selective inhibition against human PDAC cell lines [52].

**Figure 19:**
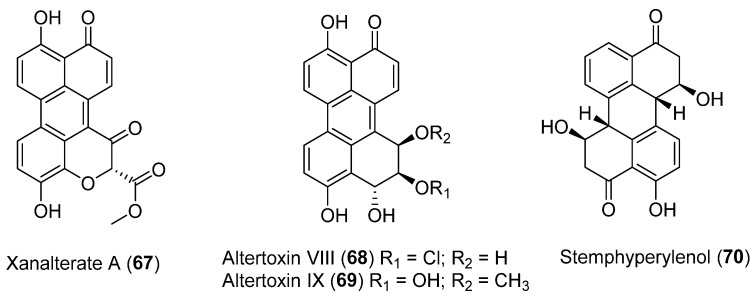
Perylenequinones isolated from *Aspergillus insulicola* HDN151418.

Three new perylenequinone derivatives (Xanalterate A, **67**, Altertoxin VIII, **68** and IX, **69**) together with a known natural product, Stemphyperylenol (**70**) (Figure 19), were isolated from *Alternaria* sp. HDN19-690 associated to an Antarctic sponge. Compound **67** exhibited promising antibacterial activity against methicillin-resistant coagulase negative *Staphylococcus* (MRCNS), *Bacillus subtilis, Proteus mirabilis, Bacillus cereus, Escherichia coli*, and *Mycobacterium phlei* with MIC values ranging from 3.13 to 12.5 µM [53].

## 4. Materials and Methods

Scifinder database and the repositories of the Pontificia Universidad Católica de Chile and Universidad Técnica Federico Santa Maria were used to search for reports published from 1996 to date. The search criteria focused on marine fungi obtained from Chilean coasts, the South Shetland Islands, and Antarctic peninsula and reports of novel marine NPs that were spectroscopically characterized and presented biological or pharmaceutical properties. Descriptions involving vegetable extracts or primary metabolites were omitted. 

## 5. Conclusions

Natural products from Chilean marine fungi represent a prolific and yet underexplored source of chemical structures with remarkable biomedical applications (Table 1). Alkaloids, polyketides, terpenoids, isoprenoids, non-isoprenoid compounds, and quinones display the most relevant biological activities. There are few studies on secondary metabolites isolated from marine fungi collected in Chile, highlighting the antimicrobial activity presented by some crude extracts and the antitumor activity of some of the isolated compounds. The dearth of studies may be attributed to the difficulties in cultivating microorganisms, some of which cannot survive under standard laboratory conditions and therefore cannot be cultured using traditional techniques. It is complicated to reproduce the conditions found inside the host marine organisms. The culture medium used is suitable for facultative fungi but probably inadequate for natural marine fungi. Recent advances in chromatographic and spectroscopic techniques now open a world of possibility for isolating secondary metabolites of these organisms that are abundant in Chilean marine ecosystems.

## Figures and Tables

**Table 1:** Secondary metabolites isolated from Chilean and Antarctic Fungi.

Compounds	Fungi	Region	Bioactivity	References
**1**	*Cladosporium cladosporioides*	Chilean coasts	Not antimicrobial activity showed	[23]
**2**	*Inonotus obliquus* *Geotrichum* sp.		Cytotoxic activity, Lipid-peroxidation, Antioxidant activity	[22,23,26]
**3**	*Penicillium brevicompactum*		Antibacterial activity	[24]
**4**				
**5**				
**6**				
**7**	*Geotrichum* sp.		Not tested	[26]
**8**	*Scleroderma polyrizum* *Geotrichum* sp. *Lampteromyces japonicus* *Fomes officinalis*		Not tested	[25,26]
**9**	*Geotrichum* sp.		Not tested	[26]
**10**	*Geomyces* sp.		Not tested	[27]
**11**	Aspergillus sp.		Antibacterial activity	[28]
**12**			Antitumor activity	
**13**			Vasorelaxant activity	
**14**	Aspergillus sp.		Not tested	[28]
**15**	*Geomyces* sp.	Antarctic	Not antimicrobial, and antifungal activity showed	[31,32,33,34]
**16**			Not antimicrobial, and antifungal activity showed	
**17**			Not antimicrobial, and antifungal activity showed	
**18**			Antifungal activity	
**19**			Antibacterial activity	
**20**	*Tridocherma asperellum*		Not tested	[35]
**21**			Not tested	
**22**			Not tested	
**23**			Not tested	
**24**			Not tested	
**25**			Not tested	
**26**	*Penicillium crustosum*		Not cytotoxic activity showed	[36,37]
**27**			Inhibit nuclear factor-κB (NF-κB)	
**28**	*Oidiodendron truncatum* GW3-13		Cytotoxic activity	[38]
**29**				
**30**				
**31**			No significant cytotoxic activity showed	
**32**			No significant cytotoxic activity showed	
**33**			No significant cytotoxic activity showed	
**34**			No significant cytotoxic activity showed	
**35**	*Pseudogymnoascus* sp.		Not antimicrobial and antifungal activity showed	[40]
**36**			Not antimicrobial and antifungal activity showed	
**37**			Not antimicrobial and antifungal activity showed	
**38**			Not antimicrobial and antifungal activity showed	
**39**	*Penicillium* sp.	Antarctic	Antiproliferative effect	[42]
**40**			Not antiproliferative effect showed	
**41**			Not antiproliferative effect showed	
**42**			Not antiproliferative effect showed	
**43**			Not antiproliferative effect showed	
**44**			Not antiproliferative effect showed	
**45**			Not antiproliferative effect showed	
**46**			Not antiproliferative effect showed	
**47**	*Penicillium crestosum* (PRB-2)		No cytotoxic and antibacterial activity showed	[44]
**48**			Antibacterial activity	
**49**			Antibacterial activity	
**50**			No cytotoxic, and antibacterial activity showed	
**51**	*Penicillium chrysogenum*		Moderate alpha glucosidase inhibition, no cytotoxic and antibacterial activity showed	[47]
**51a**			Moderate alpha glucosidase inhibition, no cytotoxic and antibacterial activity showed	
**51b**			Moderate alpha glucosidase inhibition, no cytotoxic and antibacterial activity showed	
**52a**			Moderate alpha glucosidase inhibition, no cytotoxic and antibacterial activity showed	
**52b**			Moderate alpha glucosidase inhibition, no cytotoxic and antibacterial activity showed	
**53**			Antibacterial activity	
**54**			Cytotoxic activity	
**55**			Alpha glucosidase inhibition	
**56**	*Penicillium echinulatum*		Photoprotective and antioxidant activity	[48]
**57**			No cytotoxic and antibacterial activity showed	
**58**			Cytotoxic activity	
**59**			Cytotoxic activity	
**60**	*Penicillium* sp. *CRM-1540*		Antibacterial activity	[49]
**61**	*Talaromyces* sp. HDN151403		Cytotoxic activity	[50]
**62**	*Sporothrix* sp.		Cytotoxic activity	[51]
**63**	*Aspergillus insulicola* HDN151418		No cytotoxic activity showed	[52]
**64**			No cytotoxic activity showed	
**65**			Cytotoxic activity	
**66**			Cytotoxic activity	
**67**	*Alternaria* sp. HDN19-690		Antibacterial activity, no cytotoxic activity showed	[53]
**68**			No cytotoxic and antibacterial activity showed	
**69**			No cytotoxic and antibacterial activity showed	
**70**			No cytotoxic and antibacterial activity showed	
